# Further evidence for a parent-of-origin effect at the *NOP9* locus on language-related phenotypes

**DOI:** 10.1186/s11689-016-9157-6

**Published:** 2016-06-14

**Authors:** Kerry A. Pettigrew, Emily Frinton, Ron Nudel, May T. M. Chan, Paul Thompson, Marianna E. Hayiou-Thomas, Joel B. Talcott, John Stein, Anthony P. Monaco, Charles Hulme, Margaret J. Snowling, Dianne F. Newbury, Silvia Paracchini

**Affiliations:** School of Medicine, University of St Andrews, St Andrews, KY16 9TF UK; Wellcome Trust Centre for Human Genetics, University of Oxford, Oxford, OX3 7BN UK; Worcester College, University of Oxford, Oxford, OX1 2HB UK; Department of Experimental Psychology, University of Oxford, South Parks Road, Oxford, OX1 3PT UK; Department of Psychology, University of York, York, YO10 5DD UK; School of Life and Health Sciences, Aston University, Birmingham, B4 7ET UK; Department of Physiology, University of Oxford, Parks Road, Oxford, OX1 3PT UK; Division of Psychology and Language Sciences, University College London, London, WC1 3PG UK; St John’s College, University of Oxford, Oxford, OX1 3JP UK

**Keywords:** Language impairment, Dyslexia, Genetic association, Parent-of-origin, Candidate gene

## Abstract

**Background:**

Specific language impairment (SLI) is a common neurodevelopmental disorder, observed in 5–10 % of children. Family and twin studies suggest a strong genetic component, but relatively few candidate genes have been reported to date. A recent genome-wide association study (GWAS) described the first statistically significant association specifically for a SLI cohort between a missense variant (rs4280164) in the *NOP9* gene and language-related phenotypes under a parent-of-origin model. Replications of these findings are particularly challenging because the availability of parental DNA is required.

**Methods:**

We used two independent family-based cohorts characterised with reading- and language-related traits: a longitudinal cohort (*n* = 106 informative families) including children with language and reading difficulties and a nuclear family cohort (*n* = 264 families) selected for dyslexia.

**Results:**

We observed association with language-related measures when modelling for parent-of-origin effects at the *NOP9* locus in both cohorts: minimum *P* = 0.001 for phonological awareness with a paternal effect in the first cohort and minimum *P* = 0.0004 for irregular word reading with a maternal effect in the second cohort. Allelic and parental trends were not consistent when compared to the original study.

**Conclusions:**

A parent-of-origin effect at this locus was detected in both cohorts, albeit with different trends. These findings contribute in interpreting the original GWAS report and support further investigations of the *NOP9* locus and its role in language-related traits. A systematic evaluation of parent-of-origin effects in genetic association studies has the potential to reveal novel mechanisms underlying complex traits.

## Background

Specific language impairment (SLI) is a common neurodevelopmental disorder, estimated to affect 5–10 % of preschool-aged children [1]. It is characterised by challenges with oral language acquisition, both in terms of the grammatical, syntactical and semantic aspects of speech production (expressive language) and in understanding the words of others (receptive language) [2]. An SLI diagnosis excludes other causes for language impairment such as general cognitive and neurological problems and inadequate educational opportunities [3].

A strong genetic component has been suggested by family studies, with up to one third of affected children having parents with language or literacy impairment [4]. This observation is well supported by twin studies of school-aged children, suggesting heritability to be 50 % or higher depending on diagnostic criteria [5, 6]. Linkage analyses have identified at least five SLI loci [7, 8]: 2q36 (SLI5, OMIM #615432), 7q36 (SLI4, OMIM #612514), 13q21 (SLI3, OMIM #607134), 16q24 (SLI1, OMIM #606711) and 19q13 (SLI2, OMIM #606712). Candidate genes within these regions include *CMIP* and *ATP2C2* at 16q24 [9], *CNTNAP2* at 7q36 [10] and *TM4SF20* at 2q36.3 [11].

SLI presents significant comorbidity with other neurodevelopmental disorders, particularly with dyslexia [3]. Dyslexia (reading disability; RD) is a specific impairment in learning to read, affecting approximately 10 % of children. SLI and RD present comorbidity in 43–55 % of cases [12, 13], and it is likely that shared biology contributes to both disorders. The hypothesis is supported by twin [14] and family studies [15], which indicate that common environmental and genetic influences contribute to variation in language and reading abilities. Specific genetic factors have been found to be associated with both disorders. For example, the *KIAA0319* dyslexia candidate has shown association with language skills in independent studies [16, 17]. Candidate genes for dyslexia and SLI have been shown to affect language skills in individuals with autism spectrum disorder [18].

Genome-wide association studies (GWASs) for language phenotypes have been sparse so far and have been less successful when compared to results obtained for other disorders (http://genome.gov/gwastudies/). One of the main limitations is the availability of sufficiently large and well-characterised cohorts [8]. The only significant GWAS finding for language abilities has been reported for the *ROBO2* gene through the analysis of epidemiological samples [19]. Other GWASs have used principal components of language ability within mixed-disorder cohorts but failed to detect statistically significant associations [20].

The first GWAS conducted specifically in an SLI cohort was reported recently for a cohort of 278 families [21]. Although child-based analyses did not reach significance, compelling findings were observed when modelling for parent-of-origin effects. A parent-of-origin effect is detected when an allelic influence on a trait depends on which parent a particular allele was inherited from. The most obvious explanatory mechanism would be imprinting through epigenetics. Imprinted genes are important for different aspects of brain development. Neurodevelopmental disorders, such as Prader-Willi syndrome and Angelman syndrome, which both present language deficits, involve the imprinted genomic regions on chromosome 15q (reviewed by Chamberlain and Lalande [22]). Recently, we described the first case of a deletion at the same chromosome 15 locus resulting in language impairment only, with no other syndromic manifestations [23]. It has been shown that non-imprinted genes in the mouse may still display parent-of-origin effects because of their interaction with imprinted loci and that this phenomenon is likely to be an overlooked factor contributing to complex traits [24]. The GWAS that detected parent-of-origin effects for language impairment [21] used a categorical definition of SLI based on a low score (1.5 SD, or more, below the general population mean for their age) on expressive and/or receptive subtests of the Clinical Evaluation of Language Fundamentals (CELF) [25]. A variant in *NOP9* on chromosome 14 was significantly associated (rs4280164, *P* = 3.74 × 10^−8^) with paternal parent-of-origin effects. The strongest maternal effect was observed for an intergenic variant on chromosome 5p13 but did not reach statistical significance within a GWAS context (rs10447141, *P* = 1.16 × 10^−7^). The association on chromosome 5 was followed up in a sample of 313 language-impaired children and their mothers selected from the Avon Longitudinal Study of Parents and Children (ALSPAC) cohort. While association under a maternal parent-of-origin effect was observed at this locus, the opposite allelic trend was seen. The paternal effect for *NOP9* was not followed up in the ALSPAC cohort since paternal DNA was unavailable. Replicating these associations is particularly challenging because testing parent-of-origin effects require parental DNA, which are not normally available in a case-control study design.

Here, we report the first follow-up study for the results of this GWAS for language impairment using a longitudinal cohort of 106 informative families well characterised with language-related phenotypes (332 total individuals, including parents and siblings). Given the overlap between SLI and dyslexia, we tested for association also with reading measures and analysed an additional cohort of 264 families selected primarily for dyslexia. Analysis of quantitative measures of reading and language ability, under a parent-of-origin model, showed statistically significant associations for rs4280164 at the *NOP9* locus in both cohorts but with different allelic trends and parental effects.

## Methods

### Study participants

We investigated a longitudinal cohort (referred to as the York cohort), consisting of 106 informative families characterised with reading and language measures [26]. The probands included children with a family history of dyslexia (*n* = 46), children with preschool speech and/or language difficulties (*n* = 26) and typically developing children (*n* = 41). DNA from additional family members generated a total sample size of 332 individuals. Exclusion criteria were a non-white European ethnicity and/or a non-verbal IQ score below 70. Any child with a diagnosis of Attention Deficit Hyperactivity Disorder (ADHD) was excluded from the cohort prior to sample collection. Saliva samples were extracted from children and parents using Oragene kit (prepITL2P) (DNA Genotek, Ottawa, Canada).

The York cohort included a significant proportion of children with a family history of reading difficulties in addition to children with language problems. Accordingly, we analysed an additional cohort selected for a dyslexia diagnosis. This ‘dyslexia cohort’, composed of nuclear families, has been described previously [27, 28]. Even if formal SLI diagnoses were not given to the participants of this cohort, it likely included children with language impairment given the significant comorbidity of this condition with dyslexia. The sample comprised 264 families for a total of 1037 individuals. Proband exclusion criteria included a non-white European origin, total IQ <85 and signs of other specific neurological conditions. DNA was obtained from a mix of blood and buccal swabs and extracted using standard procedures.

DNA was not available for 39 of the 212 (18 %) and 59 of the 528 (11 %) parents in the York and dyslexia cohorts, respectively.

### Phenotype selection

From the range of available language measures characterising the York cohort, we selected those most closely related to traits previously used in genetic analyses for language- and reading-related traits [17, 29–31]. These included the age at which first words are spoken (age of first words, AFW) and three core language measures from time-point 1 of the study: the ability to repeat nonwords (NWR [32]), and two expressive language measures assessed by the ability to name objects (CELFa [33]) and the ability to match spoken sentences to pictures (CELFb [33]). Three factor scores for speech (SPEECH), vocabulary (VOC) and grammar (GRAM) were also used to capture performance across language domains and were calculated for tests collected at preschool (ps) or school (sc) age. Because factor scores captured the longitudinal dimensions, all individual measures refer to the first collection in chronological time if the test was repeated at multiple times during the project. The correlation across language measures ranged between 0.37 and 0.45 for individual measures of vocabulary, grammar and phonology at time 1 (3–4 years old). Correlations between factor scores were higher; *r* = 0.8 between vocabulary and grammar at school age.

At later ages (5 ½ and 6 ½ years old), reading measures included single-word reading (READ), Test of Word Reading Efficiency (TOWRE) [34], Wechsler Individual Achievement Test spelling (WIAT-SPELL) [35], letter writing (LW), letter sound knowledge (LSK), phonological awareness measured by a phoneme isolation task (PA-is), rapid automatic naming (RAN), non-word reading (PD) and a factor score for literacy (LIT). The correlation across reading and related measures ranged between 0.5 and 0.7 at age 5 ½ years; at 6 ½ years, the correlation between reading and both the vocabulary and the grammar factor scores was 0.5 (see Hulme et al. [36] for details of the longitudinal relationships between these measures).

In contrast to the York cohort, which included longitudinal measures, the dyslexia cohort was assessed only once for study participants with an age range of 6 to 25 years and language was not measured. We analysed the reading-related measures described previously in genetic studies of this cohort [37]. This included single-word reading (READ) and spelling (SPELL), tests for orthographic coding by an irregular word task (OC-irreg) and forced choice task (OC-choice; a pseudo-homophone detection task), phoneme awareness (PA) measured by the spoonerism test, and PD. These measures show correlation with one another in the range of 0.41–0.76 [37]. The only measure directly comparable between the York and the dyslexia cohorts is READ, whereas PA, although assessing the same trait, was measured using different tests.

### Genotyping and statistical analysis

Markers rs4280164 (*NOP9*, chromosome 14) and rs10447141 (on 5p13) were genotyped using TaqMan assays (LifeTechnologies, Paisley, UK) on a ViiA7 qPCR instrument (LifeTechnologies, Paisley, UK) in the York cohort, and as part of a multiplex Sequenom assay in the dyslexia cohort. All assays passed standard quality control tests for call rate, Mendelian error and Hardy-Weinberg equilibrium. Furthermore, genome-wide genotyping data, which were available for a subset of the siblings (York, *n* = 94; dyslexia, *n* = 758), showed a 100 % concordance with genotypes generated for the entire cohorts in this study. Principal component analysis (PCA) of the genome-wide genotyping data was used to assess population stratification. All samples retained for the analysis showed no evidence of population stratification, thus allowing for a total association model, within and across families.

Statistical analysis of quantitative family data was conducted using MERLIN [38] and QTDT [39]. MERLIN was used to further control for genotyping errors and to estimate identity-by-descent (IBD) sharing scores. Parent-of-origin analysis was conducted in QTDT under the total association model (-wega -at) testing separately for maternally (-om) or paternally (-op) derived alleles and to determine whether maternal and paternal alleles were significantly different (-ot). The informative individuals used by QTDT under this model derive from two groups. The first group includes those individuals where both parents are genotyped and where one parent is homozygous or the mother and father have different genotypes. When paternal or maternal imprinting is tested, the father or the mother, respectively, must be heterozygous. The second group includes all individuals with at least one homozygous parent. The number of informative individuals in our analysis was 107 (rs10447141) and 111 (rs4280164) in the York cohort. In the dyslexia cohort, the informative individuals ranged from 443 to 470 (rs10447141) and 462 to 491 (rs4280164) depending on the amount of missing genotype or phenotypic data.

In total, we tested two markers for 25 phenotypes resulting in 50 different tests. Therefore, by applying a Bonferroni correction for multiple testing, statistical significance is reached by *P* values <0.001. This is a conservative and stringent correction as the phenotypes were correlated with one another and are not independent.

## Results

We conducted a replication study for the *NOP9* and 5p13 variants implicated in SLI susceptibility through a recent GWAS, which incorporated parent-of-origin information in the analytical model [21]. We performed quantitative association analyses with a range of language/reading-related phenotypes in two distinct cohorts. We investigated a longitudinal cohort of 315 individuals from 106 families enriched for language impairment and family history of dyslexia (York cohort). The rs4280164 and rs10447141 markers, representing the top associated variants at the *NOP9* and 5p13 loci [40], respectively, were tested for association with quantitative phenotypes. Allele frequencies (Table 1) were comparable with HapMap data for populations of European origin (HapMap minor allele frequency (MAF): rs4280164 = 18 % and rs10447141 = 29 %), although rs4280164 MAF was higher in our cohorts (22–24 %). The strongest association signal was observed between rs4280164 and age at first word (AFW) (*P* = 0.003; Table 1). The only other association was observed for rs10447141 and PA (*P* = 0.01).

**Table 1:** Association analysis result summary

Cohort	Phenotype			MAF	Standard^a^		Parent of origin			
			SNP		Risk allele	*P* value	Risk allele	*P* value -om/-op	Parental effect	*P* value -ot
	Trait	Test								
York *N* = 106 families	Language	AFW	rs4280164	A (24 %)	G	0.003	G	0.02	M	
		NWR					G	0.002		0.004
		CELFa					G	0.03		
		CELFb					G	0.02		0.009
		SPEECH (ps)					G	0.03		
		SPEECH (sc)					G	0.02		0.04
		GRAM (ps)					G	0.04		
		LW					G	0.04		
	Reading	PA-is					A	*0.001*	P	0.003
		RAN (ps)					A	0.04		0.01
	Reading	PA-is	rs10447141	T (29 %)	C	0.01				
		LSK					C	0.04	P	0.02
Dyslexia *N* = 264 families	Reading	READ	rs4280164	A (22 %)			A	0.008	M	0.03
		OC-irreg					A	*0.0004*		0.0053
		PD			A	0.045	A	0.003		0.02
		PA					A	0.01		0.03
Nudel et al. (2014)^b^ *N* = 278 probands	Language	SLI	rs4280164				G	3.74 × 10^−8^	P	
			rs10447141				C	1.16 × 10^−7^	M	

*Abbreviations*: *MAF* minor allele frequency calculated among parents, *AFW* age at first word, *NWR* non-word repetition, *CELFa* Clinical Evaluation of Language Fundamentals (expressive vocabulary), *CELFb* Clinical Evaluation of Language Fundamentals (sentence structure), *SPEECH* speech factor score, *GRAM* language grammar factor score, *LW* letter writing, *PA* phonological awareness-phoneme isolation, *RAN* rapid automatic naming, *LSK* letter sound knowledge, *READ* single-word reading, *OC-irreg* orthographic coding by an irregular word task, *ps* preschool, *sc* school, *M* maternal, *P* paternal

Only tests leading to *P* values <0.05 are reported and *P* values <0.001 (= *α* adjusted for multiple testing) are in italics

^a^Association analysis not modelled for parent of origin

^b^Analysis was conducted for a categorical definition of SLI and not for quantitative measures

When parent-of-origin was incorporated into the analysis, we observed association for a range of measures but with an inconsistent pattern. The original study reported a paternal effect at rs4280164 where the G allele was associated with an SLI status [21]. In contrast, we observed a maternal effect for the G allele across a range of language measures and a paternal effect for the A allele for some reading measures (Table 1).

In the parent-of-origin model, the association with AFW was attenuated (*P* = 0.02) but strongest associations were observed for NWR (*P* = 0.002; maternal; risk for G allele) and PA-is (*P* = 0.001; paternal; risk for A allele). The rs10447141 marker showed only marginally significant associations for LSK under a paternal origin effect (*P* = 0.04).

Given the associations we observed for PA-is in the York cohort, which included a significant proportion of children with a family history of reading difficulties, we also investigated 264 families from the ‘dyslexia cohort’. Association analysis under a standard model showed only a marginally significant association for rs4280164 with PD (*P* = 0.045).

When association analysis was conducted under a parent-of-origin model, rs4280164 showed association for most of the available reading measures with the strongest signal observed for OC-irreg (*P* = 0.0004). The trend of association was also inconsistent with previous observation; A was the risk allele under a maternal effect. No association was observed for rs10447141 with standard or parent-of-origin models in the dyslexia cohort. Paternal and maternal effects were confirmed to be statistically independent (see ‘*P* value -ot’ column in Table 1: association was detected when testing both maternal and paternal alleles in the same test) for most measures that showed parent-of-origin effects.

Although the phenotypes are correlated with each other and cannot be considered independent, our results need to be interpreted in the light of multiple testing. Two associations reached the significance level adjusted for multiple test comparisons (*α* = 0.001 for *N* = 50 tests) under a conservative assumption of independence across all tests: PA-is in the York cohort (*P* = 0.001; paternal; risk for A allele) and OC-irreg in the dyslexia cohort (*P* = 0.0004; maternal; risk for A allele).

## Discussion

We report the first follow-up analysis of a GWAS implicating two loci, one on chromosome 14 (rs4280164, *NOP9* gene) and another on chromosome 5 (rs10447141), in language impairment susceptibility under a parent-of-origin model [21]. We utilised two cohorts characterised with quantitative measures and for which parental DNA was available. The York cohort is a longitudinal study designed to investigate language and literacy development, while the dyslexia cohort was established in order to investigate the genetic component of dyslexia. The significant comorbidity between SLI and dyslexia prompted us to include reading measures in the analysis. Marker rs10447141, which did not reach statistical significance in the original study [21], only showed marginal association in the York cohort. Marker rs4280164 in the *NOP9* gene yielded significant associations when modelling for parent-of-origin across a range of measures in both cohorts but with inconsistent allelic and parental effects. The original study reported that risk was conferred by the major allele (G) with a paternal effect. Conversely, the A allele showed association in the dyslexia cohort but with a maternal effect. The results in the York cohort are intriguing: the G allele was associated with poor language skills with a maternal effect, whereas a paternal effect for the A allele was observed with some reading measures. While it would be tempting to speculate that different alleles affect different traits (e.g. reading or language) under different parental effect, caution should be used in interpreting the results until further replication analyses are conducted. It is also worth noting that opposite allelic trends were observed for the association described at the chromosome 14 loci in the two cohorts (i.e. a maternal effect was observed in both the SLI cohort and ALSPAC language-impaired subgroup but with opposite allelic effects) tested in the original study [21]. Recently, re-analysis of the SLI cohort with an improved version of the EMIM software which could support parent-of-origin analyses in a larger number of families showed a strong association for rs4280164 and decreased signal for rs10447141 in line with our findings [41]. The strong associations in the discovery study could have been an over-estimation because of the well-established phenomenon known as ‘winner’s curse’ [42], and we thus expected smaller effect sizes in this replication study.

The differences in parent-of-origin could be intrinsic to our study design and be the result of random fluctuation due to small sample sizes. The original study included 278 affected children, and the present study was based on cohorts of 106 and 264 families leading to a range of 107 to 111 and 443 to 491 informative individuals, respectively. Undoubtedly, sample size is a limitation of the present study, especially in the York cohort, which could explain the inconsistent trend. However, the strongest association was observed in the largest cohort (e.g. dyslexia cohort) for the OC-irreg phenotype (*P* = 0.0004; maternal; risk for A allele). The rs4280164 minor allele (A) has a frequency of 22–24 % (18 % reported in HapMap for European population) and corresponds to a missense variant leading to a substitution (S308N) in the protein encoded by *NOP9*. The minor allele, which gave the strongest associations in both the York and dyslexia cohorts (Table 1), was also predicted to have a damaging effect on protein function [21]. This substitution falls in a highly conserved sequence of the protein in proximity to RNA-binding domains that characterise the main function of NOP9 [21]. While a change in the protein sequence is the most obvious effect of this SNP, the rs4280164 itself is also listed as one of the most significant markers having an expression quantitative trait locus (eQTL) effect in the GTEx database (http://www.gtexportal.org/home/). An eQTL effect is reported across different tissues, the strongest of which (*P* = 3.8e–20, effect size = −0.55) is observed for the neighbouring *LTB4R* gene in the thyroid. This observation does not offer an immediate interpretation of how the SNP could affect language-related phenotype; however, it is worth noting that eQTL databases depend on the available tissues and the foetal brain, which would be the most relevant to our phenotype of interest, is not immediately accessible.

Differences in study design also have the potential to affect the results. While we tested for association with various quantitative phenotypes, the original study applied a categorical definition of SLI [21]. Further differences between the studies are represented by the use of different tests in the York and dyslexia cohorts, even when measuring the same trait. For example, phonological awareness, which showed association in both cohorts, was assessed by the phoneme isolation task in the York cohort and with the spoonerism test in the dyslexia cohort. Some of the differences in the tests used are also dependent on the age of the study participants. Participants in the York cohort were assessed mainly during preschool years, while participants in the dyslexia cohort were recruited after they started attending school and experiencing reading problems. The dyslexia cohort was not characterised with oral language measures. Lack of homogeneity for the phenotypes used in genetic studies is increasingly recognised as a major challenge in advancing the field [8].

Our findings could also reflect the complex nature of the mechanisms underlying parent-of-origin effects that can result from different phenomena. Imprinting has been demonstrated for less than 1 % human genes, but its contribution to trait variation could be higher than expected [43]. While there has not been direct evidence that *NOP9* is imprinted, it has been suggested that its location overlaps with potentially imprinted regions on chromosome 14 [41, 44, 45]. Studies that implemented parent-of-origin in their models are increasingly identifying a significant contribution of parental effects to different traits such as cancer [46], diabetes [46, 47], body mass index (BMI) [48] and pubertal timing [49]. Recently, it has been shown that an unexpectedly large number of autosomal genes (*N* = 4227) present a mono-allelic expression and a large proportion of those contribute to the variability of human traits, but *NOP9* was not tested in this study [50]. Parent-of-origin effect could be the result of an interaction between genes in imprinted region and other loci, as shown recently in mouse models [24], leading to heterogeneous and complex association patterns. Parent-of-origin evaluation might therefore explain part of the so-called missing heritability [24]. It has been consistently reported that genetic associations reported so far are only able to account for a small proportion of the estimated heritability [51]. The remaining unexplained heritability, or missing heritability, is a phenomenon observed for all complex traits and disorders, even in well-powered samples. Rare variants identified through exome and whole genome sequencing studies certainly contribute to explaining part of the missing heritability, but it is clear that other factors need to be taken into account.

Therefore, while we cannot exclude that the association patterns we report are due to chance effects, our results encourage further investigations of the NOP9 locus in the context of language abilities.

## Conclusions

In summary, we report a follow-up study for the first GWAS for SLI conducted under a parent-of-origin model by testing the top two associated markers [21]. Our study detected a parent-of-origin effect with one of these markers at the *NOP9* locus for both language and reading-related phenotypes in two independent cohorts. Although allelic and parental trends are not in line with the findings of the original GWAS, the results suggest that this locus might be implicated in neurodevelopmental phenotypes through a complex parental inheritance mechanism. To elucidate such mechanisms, it will be necessary to further study parent-of-origin effects at this locus in additional cohorts selected for reading and language impairment. Our study, once again, highlights the difficulties in conducting replication studies across heterogeneous datasets; therefore, it will be important to define common core tests that would permit direct comparison of different cohorts. We propose that parent-of-origin effects should be evaluated more systematically in association studies for language disorders as well as other complex traits since this model has the potential to elucidate some of the missing heritability.
